# New-onset lesions on MRI-DWI and cerebral blood flow changes on 3D-pCASL after carotid artery stenting

**DOI:** 10.1038/s41598-021-87339-z

**Published:** 2021-04-13

**Authors:** Wen-Xin Wang, Ting Wang, Lin Ma, Zheng-Hui Sun, Ge-Sheng Wang

**Affiliations:** 1 Department of Neurosurgery, Dongfang Hospital of Beijing University of Chinese Medicine, Fengtai District, Beijing, China; 2 Department of Radiology, Chinese PLA General Hospital, Haidian District, Beijing, China; 3 Department of Neurosurgery, Chinese PLA General Hospital, Haidian District, Beijing, China

**Keywords:** Neuroscience, Medical research, Neurology

## Abstract

This study aimed to investigate the relationship between the new-onset hyperintense lesions on diffusion-weighted images (DWI) and the changes of cerebral blood flow (CBF) before and after carotid artery stenting (CAS) in patients with symptomatic unilateral carotid artery stenosis. Twenty-four patients with symptomatic unilateral carotid stenosis (50–99%) were enrolled. Routine head magnetic resonance imaging and three-dimensional pseudo-continuous arterial spin labeling were taken 7 days before the surgery and for four consecutive days post CAS. While the incidence of new DWI lesions were high (17/24, 70.8%) and 176 lesions were observed among the 17 cases, there was only one subject showing the symptoms. The majority of the lesions were located at the cortex/subcortex of the ipsilateral frontal and parietal lobes (60.8%) with 92.6% of the lesions size being less than 3 mm. The CBFs in this area were significantly higher than that of the temporal lobe on the first 3 days post stenting (p < 0.05). No periprocedural CBF differences were observed between the two groups, however, the micro-embolism group presented decreased relative CBF in frontal and parietal lobes prior to stenting compared with the non-embolism group. The systolic blood pressure in the micro-embolism group at discharge was significantly lower than that at admission. The high incidence rate of micro-embolism in patients receiving CAS may not be the result of direct changes of hemodynamics in the brain but rather the loss of CBF regulation due to long-term hypoperfusion prior to the stenting.

## Introduction

Cerebrovascular disease is the number one cause of casualty in China^1,2^. There are approximately 2.7 million new-onset stroke patients each year which counted for one case every 12 s^3^. According to the mortality rate, one in every five deaths results from stroke, translating to one death from stroke every 21 s^2^. Approximately 70–80% of the stroke cases are ischemic stroke and a quarter of which are related to carotid artery stenosis, making carotid artery stenosis the primary cause of ischemic cerebrovascular disease^4^.

Atherosclerosis causes more than 90% of the carotid artery stenosis and carotid endarterectomy (CEA) had been the main treatment. However, carotid artery stenting (CAS) has become an alternative option to CEA given its broad application and the rapid progress in medical devices and intervention materials and techniques. It was reported that patients received CAS were prone to develop new lesions on DWI without symptoms^5,6^. But some studies showed that microembolization is associated with cognitive impairment^7,8^. Gensicke et al. reported that the new DWI lesions after CAS increased future cerebrovascular risk^9^. In this study, we investigated the relation between new-onset DWI hyperintensity and CBF changes in patients receiving CAS, intending to provide references for customized treatment to carotid artery stenosis.

## Materials and methods

### Participants

A total of 24 symptomatic unilateral carotid artery stenosis patients, 20 males and four females with average age of 62.7 ± 5.2 years old, were enrolled into People's Liberation Army General Hospital and Dongfang Hospital of Beijing University of Chinese Medicine between November 2015 and December 2019. All study protocols were approved by the Institutional Review Board of our two hospitals, and written informed consents were obtained from all patients. All methods were performed in accordance with the relevant guidelines and regulations.

Patients with moderate to severe carotid artery stenosis (≥ 50% measured according to the North America Symptomatic Carotid Endarterectomy Trial criteria)^10^ were confirmed by Digital Subtraction Angiography (DSA) and carotid duplex ultrasound. The inclusion criteria were as follows: (1) patients with symptomatic carotid artery stenosis; (2) DSA confirmed unilateral carotid artery narrowing > 50%, contralateral and posterior circulation arteries narrowing < 50%, and cerebral arteries narrowing < 50%; (3) the carotid stenosis was caused by atherosclerosis. Subjects with one of the following conditions were excluded: (1) unilateral carotid artery occlusion or bilateral carotid artery narrowing > 50%; (2) history of stroke or new-onset stroke in the past 2 weeks; (3) patients with symptomatic coronary artery disease; (4) patients with resistant hypertension and diabetes or poor blood pressure and glucose control; (5) patients received CEA due to personal preference or other underlying health conditions making CAS unfavorable; (6) patients who had poor imaging results, did not complete the study, or sign the consent.

### Magnetic resonance imaging

All subjects received magnetic resonance imaging (MRI) within seven days before the surgery (after DSA examination) and for 4 consecutive days post CAS, including T1-and T2-weighted imaging, diffusion-weighted imaging (DWI), three-dimensional fast-spoiled gradient-recalled echo sequence (3D-FSPGR), and three-dimensional pseudo-continuous arterial spin labeling (3D-pCASL). The post-labeling delay (PLD) was 2.0 s.

MRI was performed with a GE Discovery MR750 3.0 T system (GE Healthcare, Milwaukee, WI) using a 32-channel head coil. The 3D-pCASL was acquired using 3D spiral fast spin echo sequence with the following parameters: 36 slices, thickness = 4.0 mm, field of view (FOV) = 24 × 24 cm, repetition time (TR) = 4844 ms, echo time (TE) = 10.5 ms, spatial resolution = 3.64 mm, number of excitation (NEX) = 3, and the background suppression pulse was on. The parameters for 3D FSPGR were: 156bslices, thickness = 1 mm, FOV = 24 × 24 cm, TR = 8.2 ms, TE = 3.2 ms, bandwidth = 31.2 kHz, inversion time (TI) = 450 ms, matrix = 256 × 256, and NEX = 1.

The 3D-pCASL CBF images were processed by using Function Tool in Advantage Workstation 4.5 software (GE Healthcare, Milwaukee, WI) and transferred for frontal, parietal, and temporal lobes CBF quantification. The 3D FSPGR were first normalized to the Montreal Neurological Institute templates using the SPM8 software collection (Statistical Parametric Mapping, University College of London, available at www.fil.ion.ucl.ac.uk/spm/software/Spm8) in Matlab (R2013b; math Works, Natick, MA). The MR images were then coregistered to the normalized 3D FSPGR followed by a 6 mm Gaussian smoothing filter. The three-dimentional CBF measures in frontal, parietal, and temporal lobes were then abstracted with DPABI software (Data Processing & Analysis of Brain Imaging)^11–13^.

### Treatments and periprocedural events analysis

A daily dose of 100 mg aspirin and 75 mg Clopidogrel were given to all subjects at least a week before operation. Thromboelastography was routinely performed before stenting, and the operation was performed only in patients with platelet inhibition rate (induced by adenosine diphosphate receptor pathway and arachidonic acid pathway) up to standard. All procedures were performed under local anesthesia with a EV3 embolic protection device (Spider FX) except one subject received general anesthesia due to intolerance to the operation. Patients were monitored with electrocardiogram post stenting for 24 h or more depending on the circulation stability of the patients. Blood pressure was monitored and the systolic pressure was controlled within 100–130 mmHg to reduce the incidence of hyperperfusion syndrome and the impact of blood pressure fluctuation on CBF. Isoprenaline and dopamine were given if the heart rate was < 50 per minute and the systolic blood pressure (SBP) was < 90 mmHg or the mean arterial pressure (MAP) was < 50 mmHg.

The heart rate, blood pressure, dynamic changes of CBF, hyperperfusion condition and syndrome, and new-onset hyperintensity on DWI were monitored and recorded. Neurological functions were accessed in clinic or through phone follow-up by using a modified Rankin Scale (mRS) 30 days post stenting. The heart rates and blood pressures were taken four times a day for daily average. Hyperperfusion condition was defined as > 100% CBF increase post surgery and hyperperfusion syndrome was defined as clinical manifestations of migraine, ophthalmalgia, facial neuralgia, epilepsy, and focal neurological dysfunction caused by cerebral edema or hemorrhage excluding any ischemic lesions^14,15^. Continuous hypotension was defined as either SBP < 90 mmHg, MAP < 50 mmHg, or requirement of more than 6 h administration of vasoactive drugs^16^. MRI were read and analyzed by two experienced physicians and a senior physician participated to the interpretation of the images to help reach consensus in case of disagreement. New-onset DWI hyperintensity was determined as the occurrence of hyperintensity on DWI sequence postoperatively in the area where there was no hyperintensity before operation. Patients were then divided into micro-embolism and non-embolism groups based on the existence of new DWI hyperintensity and CBF and relative CBF (rCBF calculated as ipsilateral CBF/contralateral CBF) were compared between groups.

### Statistical analysis

Statistical analyses were performed by using SPSS 20.0 software. Measurement data was represented as mean ± SD. Independent sample t-test and paired-sample t-test were used to perform inter- and intra- group comparison respectively when the data were normal distributed. Rank sum test was used for non-gaussian distributed data. Categorical data was represented as percentage and comparison was performed with chi-square test. Bonferroni correction was performed when multiple statistical analyses was used. P < 0.05 was considered statistically significant.

### Ethical approval

The study design was approved by the appropriate ethics review board of Chinese PLA General Hospital and Dongfang Hospital of Beijing University of Chinese Medicine.

### Informed consent

We declare that all study participants provided informed consent.

## Results

### Patient characteristics and perioperative events

All patients successfully underwent the surgery without new-onset neurological dysfunctions. Among the two patients (8.3%) with hyperperfusion, one (4.2%) developed hyperperfusion syndrome. Six patients (25%) encountered hypotension and bradycardia. Of the 17 patients (70.8%) with new DWI lesions, one presented nausea, vomiting, vertigo, and nystagmus which were resolved in 2 days, while the rest patients exhibited no clinical symptoms (Table 1).

**Table 1:** Patient characteristics and periprocedural events in patients with or without microembolism.

	Microembolism (n = 17)	Non-embolism (n = 7)	*P* value
Age	62.4 ± 3.8	63.6 ± 6.3	0.610
Gender, female/male	2/15	2/5	0.552
Hypertension	10 (58.8%)	6 (85.7%)	0.352
Hyperlipidemia	10 (58.8%)	1 (14.3%)	0.078
Diabetes	11 (64.7%)	1 (14.3%)	0.069
Smoking	11 (64.7%)	5 (71.4%)	1.000
BMI	26.0 ± 3.8	23.6 ± 4.5	0.190
History of coronary heart disease	6 (35.3%)	3 (42.9%)	1.000
History of stroke	9 (52.9%)	5 (71.4%)	0.653
lesion side, left/right	6/11 (35.3%)	4/3 (57.1%)	0.393
**Degree of carotid artery stenosis**			
50–70%	1 (5.9%)	1 (14.3%)	0.811
70–90%	12 (70.6%)	4 (57.1%)	
> 90%	4 (23.5%)	2 (28.6%)	
Modified Rankin scale on admission	1.47 ± 0.62	2.00 ± 0.58	0.067
Modified Rankin scale 30 days post stenting	1.24 ± 0.44	1.29 ± 0.49	0.806
Hypotension post stenting	5 (29.4%)	1 (14.3%)	0.629
Bradycardia post stenting	3 (17.6%)	3 (42.9%)	0.307
Hyperperfusion	1 (5.9%)	1 (14.3%)	0.507
Hyperperfusion syndrome	1 (5.9%)	0	1.000
Perioperative infarction	1 (5.9%)	0	1.000

There were no significant differences in demographic characteristics between the micro-embolism and non-embolism groups. Clinical characteristics including hypertension, hyperlipidemia, diabetes, smoking habits, BMI, history of cardiovascular diseases and stokes, lesion side, degree of stenosis, and mRS upon admission and discharge did not differ substantially between the two groups (Table 1).

### Analysis of new-onset DWI hyperintensity in CAS patients

A total of 176 new DWI lesions were observed in the 17 patients with hyperintense DWI after stenting. Most lesions (85.8%) appeared in the first day post CAS with 96.6% ipsilateral lesions and 92.6% lesion size of less than 3 mm. The lesions located mainly in the cerebral cortex and subcortex with 60.8% occurrences at frontal and parietal lobes and 5.7% and 13.6% at temporal lobe and occipital lobe respectively. We also observed new DWI lesions at centrum semiovale (5.7%), lateral ventricle white matter (6.8%), basal ganglia (1.1%), insula (0.6%), ipsilateral cerebellum (2.3%), and cortical watershed (33.0%) (Table 2).

**Table 2:** Distribution of new DWI lesions in patients receiving CAS.

	Number
**Location**	
Frontal lobe, cortex/subcortex	63 (35.8%)
Parietal lobe, cortex/subcortex	44 (25%)
Temporal lobe, cortex/subcortex	10 (5.7%)
Occipital lobe, cortex/subcortex	24 (13.6%)
Centrum semiovale	10 (5.7%)
Lateral ventricles	12 (6.8%)
Basal ganglia	2 (1.1%)
Cerebellum	4 (2.3%)
Insula	1 (0.6%)
Contralateral sites	6 (3.4%)
**Size**	
< 3 mm	163 (92.6%)
≥ 3 mm	13 (7.4%)
Incidences on 1 days	151 (85.8%)
Cortical watershed	58 (33.0%)

### Changes in heart rates and blood pressure post stenting

The patients in the two groups showed comparable heart rates in baseline and post-stenting statuses, despite the observation of reduced heart rates on the first 2 days after the operations and a return to normal rates gradually afterward. There were no differences in systolic and diastolic blood pressures between groups upon admission and discharge. While the micro-embolism group showed significantly lower systolic blood pressure at discharge admission than that on admission (136.5 ± 18.0 vs 124.9 ± 14.9, P < 0.05), the non-embolism group presented lower diastolic blood pressure at discharge admission than that on admission (81.1 ± 10.4 vs 69.6 ± 9.8, P < 0.05).

### Dynamic changes in CBF post stenting

Cerebral blood flows in the frontoparietal and temporal lobes were measured to further investigate the relation between CBF dynamics and the formation of embolism after stenting. All patients exhibited comparable pre-surgery CBFs in these two locations and experienced significant higher CBFs in both ipsilateral and contralateral frontoparietal lobes on day 1–3 post stenting compared with that of temporal lobe. The differences disappeared on day 4. We also observed increased ipsilateral CBFs from baselines in these two locations for 4 consecutive days after the surgery with most patients’ CBF peaked at day 3 before it decreased (Table 3, Supplementary file).

**Table 3:** The cerebral blood flows (mL/100 g/min) in frontoparietal and temporal lobes of patients receiving CAS.

	Frontoparietal lobes		Temporal lobe		*P* value	
	Ipsilateral (L1)	Contralateral (C1)	Ipsilateral (L2)	Contralateral (C2)	L1 v.s L2	C1 v.s C2
Pre-surgery (D0)	39.10 ± 4.31	44.08 ± 4.68	38.44 ± 4.62	42.72 ± 4.15	0.075	0.161
Day 1 post stenting (D1)	46.93 ± 7.88	45.69 ± 4.85	44.36 ± 6.53	43.34 ± 4.98	0.001	0.006
Day 2 post stenting (D2)	48.23 ± 9.07	47.89 ± 6.28	46.65 ± 8.27	45.75 ± 6.67	0.018	0.011
Day 3 post stenting (D3)	50.49 ± 8.02	50.16 ± 5.79	47.94 ± 9.08	48.00 ± 5.98	0.011	0.000
Day 4 post stenting (D4)	45.58 ± 9.37	45.48 ± 6.50	43.91 ± 7.80	43.67 ± 6.28	0.107	0.067

When patients were grouped according to the presence of new DWI lesions, we did not see CBF differences in the frontoparietal and temporal lobes between the two groups despite the patients exhibited significant increased ipsilateral CBFs post intervention (Tables 4 and 5, Supplementary file).

**Table 4:** The cerebral blood flows (mL/100 g/min) in frontoparietal lobes of patients with or without microembolism.

	Microembolism		Non-embolism		*P* value	
	Ipsilateral (L1)	Contralateral (C1)	Ipsilateral (L2)	Contralateral (C2)	L1 v.s L2	C1 v.s C2
Pre-surgery (D0)	38.82 ± 2.32	44.77 ± 4.80	39.76 ± 7.49	42.39 ± 4.24	0.757	0.267
Day 1 post stenting (D1)	46.05 ± 8.74	45.00 ± 5.30	49.07 ± 5.18	42.39 ± 4.24	0.405	0.282
Day 2 post stenting (D2)	46.83 ± 10.13	47.76 ± 6.72	51.63 ± 4.78	48.19 ± 5.52	0.247	0.885
Day 3 post stenting (D3)	49.59 ± 8.91	49.83 ± 6.49	52.67 ± 5.21	50.98 ± 3.88	0.406	0.669
Day 4 post stenting (D4)	44.45 ± 10.50	45.27 ± 7.31	48.32 ± 5.47	45.97 ± 4.39	0.370	0.815

**Table 5:** The cerebral blood flows (mL/100 g/min) in temporal lobe of patients with or without microembolism.

	Microembolism		Non-embolism		*P* value	
	Ipsilateral (L1)	Contralateral (C1)	Ipsilateral (L2)	Contralateral (C2)	L1 v.s L2	C1 v.s C2
Pre-surgery (D0)	38.08 ± 2.99	41.97 ± 3.84	39.32 ± 7.54	44.55 ± 4.59	0.686	0.171
Day 1 post stenting (D1)	43.25 ± 7.08	43.45 ± 5.02	47.06 ± 4.26	43.09 ± 5.27	0.202	0.878
Day 2 post stenting (D2)	45.03 ± 8.88	45.55 ± 6.99	50.59 ± 5.11	46.25 ± 6.31	0.138	0.820
Day 3 post stenting (D3)	46.85 ± 10.30	47.51 ± 6.47	50.58 ± 4.65	49.21 ± 4.79	0.372	0.538
Day 4 post stenting (D4)	42.76 ± 8.42	42.78 ± 6.64	46.68 ± 5.61	45.83 ± 5.12	0.272	0.290

The relative CBF (rCBF) in the frontoparietal and temporal lobes were further compared between groups and we observed a significant reduced rCBF of the frontoparietal lobes in the micro-embolism group (0.861 in the microembolism group vs 0.912 in the non-embolism group, P < 0.05), whereas the rCBF of temporal lobe showed no differences between the two groups (0.975 in the microembolism group vs 0.888 in the non-embolism group, P > 0.05).

## Discussion

Several large-scale randomized clinical trials have reported that patients receiving CAS have greater risk of stroke than CEA patients within 30 days post surgery^17–20^. The differences of perioperative stroke are the results of excessive mild non-disabling infarctions. Currently recognized possible mechanisms of ischemic stroke involve hemodynamics mechanism, artery-to-artery embolism, and combination of the two. To investigate the pathogenetic mechanism of perioperative microembolism, we analyzed the size and distribution of the DWI lesions and changes of hemodynamics in patients receiving CAS.

In this study, we showed 17 (70.8%) patients with new DWI lesions and most of them were free from symptoms, except one patient experienced vertigo and nystagmus. The new lesions on DWI images are generally considered as embolism resulting from plaque rupture or dislodged during the stenting procedure^5,6^. Other studies also indicated that it can be a result of continuous emboli dislodging after the surgery, although the cause and mechanism are still not clear^21^^.^

While the majority of perioperative micro-embolism occurred as ipsilateral hemispheric events with a few were located in areas unrelated to the carotid artery therapies^10,22^, contralateral micro-emboli after intervention may be caused by catheter destruction of the aortic arch plaques^22^. Our study showing contralateral embolism observable 1 day after surgery is in keeping with the mechanism of catheter induced thrombosis. Although 85.8% of the emboli were observed on day 1 post stenting, the discovery of new lesions on day 2–4 indicated a continuous spontaneous release of unstable emboli.

The cerebral cortical arteries, also known as pial collaterals, are small arterial networks joining the terminal branches of anterior, middle, and posterior cerebral arteries along the surface of the brain. The vessel branches penetrate through the cortex, white matter, and the axon fibers with the deepest ones forming the perforating medullary branches supplying the blood to centrum semiovale. The medullary branches locate at the distal end of the internal carotid artery with a relatively low perfusion pressure. As the medullary branches rarely anastomose with the deep perforating branches, hypoperfusion is more prone to occur in centrum semiovale in the presence of severe stenosis of internal carotid artery. It has been shown that large particles may be restricted from entering these blood vessels, however, particles less than 150 μm show equal opportunity of entry^23,24^. The less micro-embolic incidences in this area might be the result of relative low blood flows compared with the cortex^25^. Our study showed that the majority (60.8%) of micro-embolism occurred at the cortex/subcortex of the frontal and parietal lobes, and only 5.7% and 6.8% were observed in centrum semiovale and paraventricular white matter, respectively. In addition, the fact that these micro-embolism did not mainly occur in the cortical watershed territory, suggested the hypoperfusion might not be the causal mechanism of micro-embolism. This view is further strengthened by our observation of reduced cortical CBF and incidences of embolism in temporal lobe post stenting compared with that of the frontal and parietal lobes. The less increase of CBF in temporal lobe can be partially explained by the disproportionate increment of blood flow after stenting as a portion of the temporal lobe, particularly the basal temporal region, is supplied by the posterior cerebral artery.

We further grouped the patients into non-embolism and micro-embolism groups based on the presence of new DWI lesions after intervention. Interestingly, we observed a significantly reduced rCBF in the frontal and parietal lobes prior to the surgery in the micro-embolism group, but the basal and post stenting CBFs of the 2 groups were comparable. The relative CBF is associated with impaired regulation of pre-operative CBF, indicating a possible relationship between micro-embolism and impaired CBF regulation caused by long-term hypoperfusion in patients receiving CAS. This phenomenon was not observed in the temporal lobe, probably because it is fed by anterior and posterior circulations whereas the frontal and parietal lobes are fed by anterior circulation only.

When the catheter passed the unstable plaque, thousands of the emboli were released and circulated along the edge of the blood vessel wall^25,26^. While most of the emboli penetrated through the vessel wall^27^, a few arrived at the corresponding arteries on the surface of the brain. The blood vessels may be capable of regulating the vasodilation for maintaining the blood supply in the local brain tissue until the emboli are dissolved or expelled. However, ischemia is inevitable when the arteries are in its critical condition and lose the ability of regulating blood supply due to long-term hypoperfusion^27^. Because of the higher CBF in the frontal and parietal lobes, the microemboli mainly flow to and lodge in these areas. Further research is needed to support our speculation.

Although most micro-embolism do not cause apparent neurological dysfunction, some studies have shown otherwise^7,8^. Zhou et al. reported a correlation between the volume of subclinical embolic infarct and long-term cognitive changes after carotid revascularization^28^. Other animal studies also demonstrated neural and cognitive damages in association with the size of microemboli^29,30^. Thus, it is of important to further compare the changes of long-term cognitive function between patients receiving CAS and CEA.

In this study, we could see increased CBF in contralateral hemisphere after stenting of ipsilateral side. For carotid artery stenosis may lead to ipsilateral cerebral hemisphere ischemia, contralateral CBF may supply this area due to the existence of circle of Willis. Similarly, the ipsilateral CBF can flow to the contralateral cerebral hemisphere after stenting through the circle of Willis, which increases the contralateral CBF.

We observed that there was no difference in systolic and diastolic blood pressure between the two groups at admission and discharge. But in the microembolism group, the systolic blood pressure at discharge was significantly lower than that at admission. We speculate that patients with impaired CBF regulation are more likely to develop microembolism if their systolic blood pressure drops too much after operation. This result needs to be confirmed by further research.

We recognize the limitations of this study as follows: (1) the relative small sample size; (2) we did not analyze the risk factors of the micro-embolism; (3) we did not measure the patients’ cognition; (4) some patients did not show up for the follow-up MRI examination 30 days after the surgery; (5) we performed a relatively short-term follow-up as most subjects came from other provinces.

## Conclusion

The CAS-induced micro-embolism might not be the result of direct changes of hemodynamics in the brain but rather the loss of CBF regulation due to long-term hypoperfusion prior to the stenting. It might also be related to the excessive decrease of systolic blood pressure post stenting.

## Supplementary Information

Supplementary Information.

## References

[1] Zhou M, Wang H, Zhu J (2016). Cause-specific mortality for 240 causes in China during 1990–2013: A systematic subnational analysis for the global burden of disease study 2013. Lancet (Lond., Engl.).

[2] Wu S, Wu B, Liu M (2019). Stroke in China: Advances and challenges in epidemiology, prevention, and management. Lancet Neurol..

[3] Wang L (2015). Report on Prevention and Treatment of Stroke in China.

[4] Kastrup A, Groschel K, Nagele T (2008). Effects of age and symptom status on silent ischemic lesions after carotid stenting with and without the use of distal filter devices. AJNR Am. J. Neuroradiol..

[5] Poppert H, Wolf O, Theiss W (2006). MRI lesions after invasive therapy of carotid artery stenosis: A risk-modeling analysis. Neurol. Res..

[6] Flach HZ, Ouhlous M, Hendriks JM (2004). Cerebral ischemia after carotid intervention. J. Endovasc. Therapy.

[7] Maggio P, Altamura C, Landi D (2013). Diffusion-weighted lesions after carotid artery stenting are associated with cognitive impairment. J. Neurol. Sci..

[8] Hitchner E, Baughman BD, Soman S, Long B, Rosen A, Zhou W (2016). Microembolization is associated with transient cognitive decline in patients undergoing carotid interventions. J. Vasc. Surg..

[9] Gensicke H, van der Worp HB, Nederkoorn PJ (2015). Ischemic brain lesions after carotid artery stenting increase future cerebrovascular risk. J. Am. Coll. Cardiol..

[10] Barnett HJM, Taylor DW, Haynes RB (1991). Beneficial effect of carotid endarterectomy in symptomatic patients with high-grade carotid stenosis. N. Engl. J. Med..

[11] Nowinski WL, Qian G, Kirgaval Nagaraja BP (2006). Analysis of ischemic stroke MR images by means of brain atlases of anatomy and blood supply territories. Acad. Radiol..

[12] Lee JS, Lee DS, Kim YK (2004). Probabilistic map of blood flow distribution in the brain from the internal carotid artery. Neuroimage.

[13] Zhu J, Zhuo C, Qin W (2015). Altered resting-state cerebral blood flow and its connectivity in schizophrenia. J. Psychiatr. Res..

[14] Ogasawara K, Sakai N, Kuroiwa T (2007). Intracranial hemorrhage associated with cerebral hyperperfusion syndrome following carotid endarterectomy and carotid artery stenting: Retrospective review of 4494 patients. J. Neurosurg..

[15] van Mook WN, Rennenberg RJ, Schurink GW (2005). Cerebral hyperperfusion syndrome. Lancet Neurol..

[16] Trocciola SM, Chaer RA, Lin SC (2006). Analysis of parameters associated with hypotension requiring vasopressor support after carotid angioplasty and stenting. J. Vasc. Surg..

[17] Ederle J, Dobson J, Featherstone RL (2010). Carotid artery stenting compared with endarterectomy in patients with symptomatic carotid stenosis (International Carotid Stenting Study): An interim analysis of a randomised controlled trial. Lancet (Lond., Engl.).

[18] Mas JL, Trinquart L, Leys D (2008). Endarterectomy versus angioplasty in patients with symptomatic severe carotid stenosis (EVA-3S) trial: Results up to 4 years from a randomised, multicentre trial. Lancet Neurol..

[19] Ringleb PA, Allenberg J, Bruckmann H (2006). 30 day results from the SPACE trial of stent-protected angioplasty versus carotid endarterectomy in symptomatic patients: A randomised non-inferiority trial. Lancet (Lond., Engl.).

[20] Brott TG, Hobson RW, Howard G (2010). Stenting versus endarterectomy for treatment of carotid-artery stenosis. N. Engl. J. Med..

[21] Rapp JH, Wakil L, Sawhney R (2007). Subclinical embolization after carotid artery stenting: New lesions on diffusion-weighted magnetic resonance imaging occur postprocedure. J. Vasc. Surg..

[22] Huibers A, Calvet D, Kennedy F (2015). Mechanism of procedural stroke following carotid endarterectomy or carotid artery stenting within the international carotid stenting study (ICSS) randomised trial. Eur. J. Vasc. Endovasc. Surg..

[23] Pollanen MS, Deck JH (1990). The mechanism of embolic watershed infarction: Experimental studies. Can. J. Neurol. Sci..

[24] Macdonald RL, Kowalczuk A, Johns L (1995). Emboli enter penetrating arteries of monkey brain in relation to their size. Stroke.

[25] Zhu L, Wintermark M, Saloner D, Fandel M, Pan XM, Rapp JH (2011). The distribution and size of ischemic lesions after carotid artery angioplasty and stenting: Evidence for microembolization to terminal arteries. J. Vasc. Surg..

[26] Bushi D, Grad Y, Einav S, Yodfat O, Nishri B, Tanne D (2005). Hemodynamic evaluation of embolic trajectory in an arterial bifurcation: An in-vitro experimental model. Stroke.

[27] Lam CK, Yoo T, Hiner B, Liu Z, Grutzendler J (2010). Embolus extravasation is an alternative mechanism for cerebral microvascular recanalization. Nature.

[28] Zhou W, Baughman BD, Soman S (2017). Volume of subclinical embolic infarct correlates to long-term cognitive changes after carotid revascularization. J. Vasc. Surg..

[29] Nemeth CL, Shurte MS, McTigue DM, Nemeroff CB, Neigh GN (2012). Microembolism infarcts lead to delayed changes in affective-like behaviors followed by spatial memory impairment. Behav. Brain Res..

[30] Rapp JH, Pan XM, Neumann M, Hong M, Hollenbeck K, Liu J (2008). Microemboli composed of cholesterol crystals disrupt the blood-brain barrier and reduce cognition. Stroke.

