# Author Correction: New-onset lesions on MRI-DWI and cerebral blood flow changes on 3D-pCASL after carotid artery stenting

**DOI:** 10.1038/s41598-022-06550-8

**Published:** 2022-02-08

**Authors:** Wen-Xin Wang, Ting Wang, Lin Ma, Zheng-Hui Sun, Ge-Sheng Wang

**Affiliations:** 1 Department of Neurosurgery, Dongfang Hospital of Beijing University of Chinese Medicine, Fengtai District, Beijing, China; 2 Department of Radiology, Chinese PLA General Hospital, Haidian District, Beijing, China; 3 Department of Neurosurgery, Chinese PLA General Hospital, Haidian District, Beijing, China

Correction to: *Scientific Reports* https://doi.org/10.1038/s41598-021-87339-z, published online 13 April 2021

The original version of this Article contained an error in the author list. Xin Lou was incorrectly listed as an author of the original Article, and has subsequently been removed.

The Acknowledgments section now reads:

The authors would like to thank Professor Lou Xin for her technical guidance.

The Author Contributions section now reads:

W.W. collected data and wrote the paper. T.W. and Z.S. performed data analysis. L.M., and G.W. conceived the research, provided guidance, discussed the data, revised and improved the paper.

The Funding section “This study was funded by the National Natural Science Foundation of China (Contract Grant Numbers: 81671126 to X.L.).” has been removed.

The original Article and accompanying Supplementary Information file have been corrected.

