# Supplementary material for: New-onset lesions on MRI-DWI and cerebral blood flow changes on 3D-pCASL after carotid artery stenting
Source: Sci Rep. 2021 Apr 13;11:8005. doi: 10.1038/s41598-021-87339-z (PMC8044121; doi:10.1038/s41598-021-87339-z)
Supplement: Supplementary file 1 — Supplementary Information. [file 41598_2021_87339_MOESM1_ESM.docx]

New-onset Lesions on MRI-DWI and Cerebral Blood Flow Changes on 3D-pCASL after Carotid Artery Stenting

Wen-Xin Wang^1,2^, MD, PhD, Ting Wang^2^, MD, Lin Ma^2^ MD, PhD, Zheng-Hui Sun^3^, MD, PhD, Ge-Sheng Wang^1^, MD, PhD

^1^Department of Neurosurgery, Dongfang Hospital of Beijing University of Chinese Medicine, Fengtai District, Beijing, China

^2^Department of Radiology, Chinese PLA General Hospital, Haidian District, Beijing, China

^3^Department of Neurosurgery, Chinese PLA General Hospital, Haidian District, Beijing, China

Corresponding author: Professor Gesheng Wang; Tel.: +86 13581759316; fax: +86 010 67689735. Email address: [shoushudao0416@sohu.com](mailto:shoushudao0416@sohu.com)

Table 1. The cerebral blood flows (mL/100g/min) in frontoparietal and temporal lobes of patients receiving CAS.

|  | | Pre-surgery (D0) | Day 1 post stenting (D1) | Day 2 post stenting (D2) | Day 3 post stenting (D3) | Day 4 post stenting (D4) | *P* value | | | |
| --- | --- | --- | --- | --- | --- | --- | --- | --- | --- | --- |
|  |  |  |  |  |  |  | D0 v.s D1 | D0 v.s D2 | D0 v.s D3 | D0 v.s D4 |
| Frontoparietal lobes | Ipsilateral | 39.10±4.31 | 46.93±7.88 | 48.23±9.07 | 50.49±8.02 | 45.58±9.37 | <0.0125 | <0.0125 | <0.0125 | <0.0125 |
|  | Contralateral | 44.08±4.68 | 45.69±4.85 | 47.89±6.28 | 50.16±5.79 | 45.48±6.50 | >0.0125 | <0.0125 | <0.0125 | >0.0125 |
| Temporal lobe | Ipsilateral | 38.44±4.62 | 44.36±6.53 | 46.65±8.27 | 47.94±9.08 | 43.91±7.80 | <0.0125 | <0.0125 | <0.0125 | <0.0125 |
|  | Contralateral | 42.72±4.15 | 43.34±4.98 | 45.75±6.67 | 48.00±5.98 | 43.67±6.28 | >0.0125 | <0.0125 | <0.0125 | >0.0125 |

Table 2. The cerebral blood flows (mL/100g/min) in frontoparietal lobes of patients with or without microembolism.

|  | | Pre-surgery (D0) | Day 1 post stenting (D1) | Day 2 post stenting (D2) | Day 3 post stenting (D3) | Day 4 post stenting (D4) | *P value* | | | |
| --- | --- | --- | --- | --- | --- | --- | --- | --- | --- | --- |
|  |  |  |  |  |  |  | D0 v.s D1 | D0 v.s D2 | D0 v.s D3 | D0 v.s D4 |
| Microembolism | Ipsilateral | 38.82±2.32 | 46.05±8.74 | 46.83±10.13 | 49.59±8.91 | 44.45±10.50 | <0.0125 | <0.0125 | <0.0125 | >0.0125 |
|  | Contralateral | 44.77±4.80 | 45.00±5.30 | 47.76±6.72 | 49.83±6.49 | 45.27±7.31 | >0.0125 | >0.0125 | <0.0125 | >0.0125 |
| Non-embolism | Ipsilateral | 39.76±7.49 | 49.07±5.18 | 51.63±4.78 | 52.67±5.21 | 48.32±5.47 | >0.0125 | <0.0125 | <0.0125 | >0.0125 |
|  | Contralateral | 42.39±4.24 | 42.39±4.24 | 48.19±5.52 | 50.98±3.88 | 45.97±4.39 | <0.0125 | <0.0125 | <0.0125 | >0.0125 |

Table 3. The cerebral blood flow (mL/100g/min) in temporal lobe of patients with or without microembolism.

|  | | Pre-surgery (D0) | Day 1 post stenting (D1) | Day 2 post stenting (D2) | Day 3 post stenting (D3) | Day 4 post stenting (D4) | *P* value | | | |
| --- | --- | --- | --- | --- | --- | --- | --- | --- | --- | --- |
|  |  |  |  |  |  |  | D0 v.s D1 | D0 v.s D2 | D0 v.s D3 | D0 v.s D4 |
| Microembolism | Ipsilateral | 38.08±2.99 | 43.25±7.08 | 45.03±8.88 | 46.85±10.30 | 42.76±8.42 | <0.0125 | <0.0125 | <0.0125 | >0.0125 |
|  | Contralateral | 41.97±3.84 | 43.45±5.02 | 45.55±6.99 | 47.51±6.47 | 42.78±6.64 | >0.0125 | >0.0125 | <0.0125 | >0.0125 |
| Non-embolism | Ipsilateral | 39.32±7.54 | 47.06±4.26 | 50.59±5.11 | 50.58±4.65 | 46.68±5.61 | <0.0125 | <0.0125 | >0.0125 | >0.0125 |
|  | Contralateral | 44.55±4.59 | 43.09±5.27 | 46.25±6.31 | 49.21±4.79 | 45.83±5.12 | >0.0125 | >0.0125 | <0.0125 | >0.0125 |
